# ExtRamp: a novel algorithm for extracting the ramp sequence based on the tRNA adaptation index or relative codon adaptiveness

**DOI:** 10.1093/nar/gky1193

**Published:** 2019-01-15

**Authors:** Justin B Miller, Logan R Brase, Perry G Ridge

**Affiliations:** Department of Biology, Brigham Young University, Provo, UT 84602, USA

## Abstract

Different species, genes, and locations within genes use different codons to fine-tune gene expression. Within genes, the ramp sequence assists in ribosome spacing and decreases downstream collisions by incorporating slowly-translated codons at the beginning of a gene. Although previously reported as occurring in some species, no previous attempt at extracting the ramp sequence from specific genes has been published. We present ExtRamp, a software package that quickly extracts ramp sequences from any species using the tRNA adaptation index or relative codon adaptiveness. Different filters facilitate the analysis of codon efficiency and enable identification of genes with a ramp sequence. We validate the existence of a ramp sequence in most species by running ExtRamp on 229 742 339 genes across 23 428 species. We evaluate differences in reported ramp sequences when we use different parameters. Using the strictest ramp sequence cut-off, we show that across most taxonomic groups, ramp sequences are approximately 20–40 codons long and occur in about 10% of gene sequences. We also show that in *Drosophila melanogaster* as gene expression increases, a higher proportion of genes have ramp sequences. We provide a framework for performing this analysis on other species. ExtRamp is freely available at https://github.com/ridgelab/ExtRamp.

## INTRODUCTION

The central dogma of biology shows that three consecutive nucleotides of coding DNA, called codons, are transcribed into messenger RNA (mRNA), mRNA is translated into amino acids, and amino acids form proteins (1). There are 61 canonical codons plus three stop codons that form and regulate the creation of 20 amino acids (2). Since there are more codons than amino acids, in many cases multiple synonymous codons encode the same amino acid. Although originally presumed to be identical in function, unequal distributions of synonymous codons quickly led to two non-mutually exclusive hypotheses: (i) non-random mutations occur particularly at the third codon position, and (ii) selection for codon bias persists (3,4). Furthermore, highly expressed genes display more prominent codon usage biases, suggesting that synonymous codons might play different roles in species fitness (5). The unequal abundance of tRNA anticodons led to the wobble hypothesis: tRNA anticodons do not need to latch onto all three codon nucleotides (6). However, codon usage is highly associated with the most abundant tRNA present in the cell (7). Furthermore, codon usage patterns affect gene expression, with codons latching onto fewer than all three tRNA anticodons being considered suboptimal for gene expression (8).

Although increased gene expression is often considered optimal, suboptimal codons are preferred in certain genes or parts of genes because they slow translation and reduce translational errors. For instance, a short set of 30–50 slowly-translated suboptimal codons, or ramp sequence, was identified at the 5′ end of many protein coding sequences, which serves to evenly space ribosomes (9) and reduce mRNA secondary structure (10) at translation initiation. This region has codons that are less adapted to the tRNA pool and consequently the ramp sequence has a slower elongation speed relative to the rest of the gene (11). This ramp could be caused by any of three features correlating with slower translation elongation speed: codon adaptation to the tRNA pool, amino acid charge, and mRNA folding energy (12). The ramp sequence was discovered by using a sliding window of 15 codons (although verification was done with sliding windows ranging from 10 to 20 codons), representing the length of the ribosome footprint (9). However, more recent estimates of the ribosome footprint range from 15 nucleotides (5 codons) to about 45 nucleotides (15 codons) with a commonly accepted length of 28 nucleotides (about 9 codons) (13). Therefore, any algorithm for extracting the ramp sequence must be capable of adapting to different ribosome footprints by changing the size of the sliding window. Finally, since the ramp sequence is a relative measure of codon efficiency for each gene and not an absolute measure for the whole genome, each gene sequence must be analyzed individually (11).

Several methods have been used to calculate the effect that each codon has on overall translation efficiency. Two of the most common approaches are the *Codon Adaptation Index* (CAI) (14), which calculates a normalized value for each codon based on a set of highly expressed genes from the organism, and the *Effective Number of Codons* (N_c_) model (15), which uses a population genetics approach to calculate the efficiency of each codon based on its overall usage in the species. To calculate CAI, two relative adaptiveness measures are used. First, the relative synonymous codon usage (RSCU) is calculated by dividing the observed frequency of a codon by the frequency of each codon encoding the same amino acid, assuming equal usage. Second, the relative adaptiveness of a codon (*w_ij_*) is calculated for the *j*th codon in the *i*th amino acid. The *w_ij_* metric is the ratio of RSCU_*ij*_ to RSCU_imax_ for the *i*th amino acid (14). The *tRNA Adaptation Index* (tAI) (16) more accurately reflects changes in overall translational efficiency due to wobble interactions, tRNA composition, and synonymous codon position within a gene (17). However, most species do not have annotated tAI values. Since the tAI and *w*_*ij*_ both measure overall translational efficiency, *w*_*ij*_ can be used as a proxy for tAI when only sequence data are available.

We present ExtRamp, the first algorithm that can identify areas of decreased translational efficiency at the start of individual genes using tAI, *w_ij_* or any other codon efficiency table. No existing algorithm can identify ramp sequences in individual genes. We validate our approach by recreating the whole genome trends identified by Tuller *et al.* (9) in *Saccharomyces cerevisiae, Drosophila melanogaster* and *Caenorhabditis elegans* using tAI values. Moreover, we demonstrate the effectiveness of *w*_*ij*_ as proxy for tAI by using it to detect the same pattern. Finally, we provide statistics of ramp usages and relative codon adaptiveness in 23 428 species across all domains of life.

## MATERIALS AND METHODS

### Data collection and processing

We use the coding sequences (CDS) from 23 428 species from the following taxonomic groups with some overlap between viruses and bacteria: 418 archaea, 15 063 bacteria, 234 fungi, 149 invertebrates, 89 plants, 75 protozoa, 107 mammalian vertebrates, 123 other vertebrates, and 7 233 viruses. All CDS regions were downloaded from the National Center for Biotechnology Information (NCBI) in September 2017 (18–20). The reference sequences for each gene were used because they are the most complete compilation of the alleles in a given species (20). We always used the longest isoform, when given a choice, and we filtered out partial gene sequences and sequences with annotated exceptions (i.e. unclassified transcription discrepancy, suspected errors, translational exception, etc.).

The tAI values were downloaded from the tAI Calculator (http://tau-tai.azurewebsites.net) (21). We provide tAI data for *Escherichia coli* and *S. cerevisiae* in the GitHub repository as examples of the two comma-separated values (CSV) file formats accepted by ExtRamp. Since tAI values reflect the overall translational efficiency of a species better than *w_ij_*, we recommend using tAI values, where available.

### Extracting the ramp sequence

**Figure 1.**
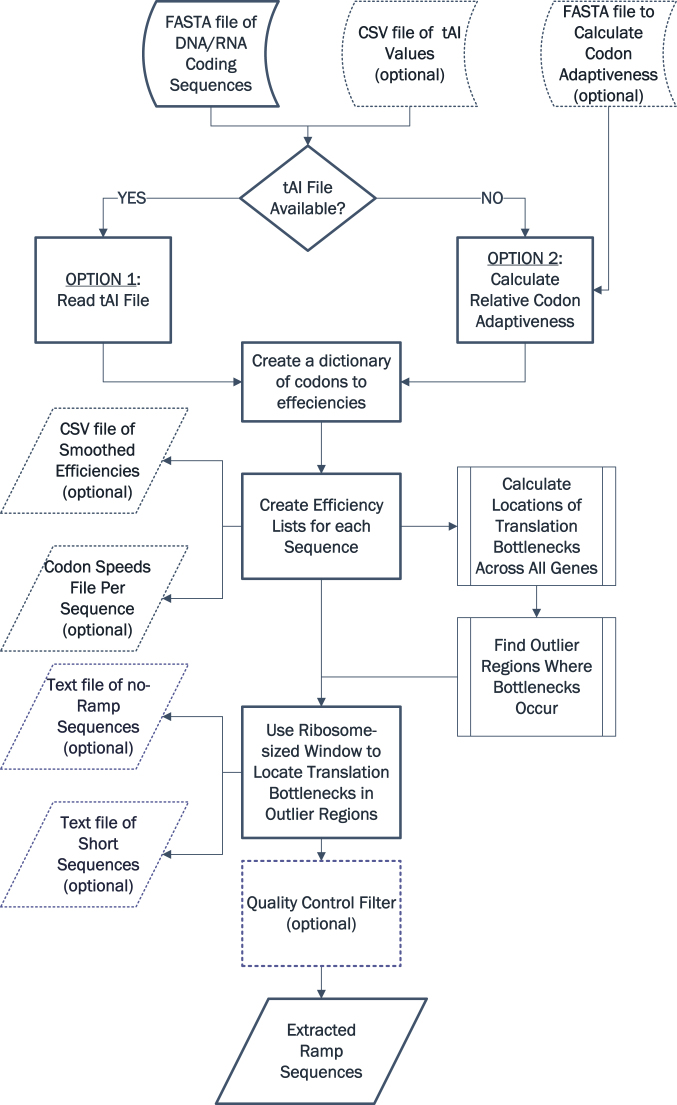
ExtRamp algorithm flowchart. Outlines the algorithm steps, including the optional input/output arguments (dotted lines) and the two options (underlined) for calculating codon efficiencies.

ExtRamp has two options to extract the ramp sequence, determined by user input (see Figure 1). The first option uses tAI values (or other codon efficiency values). ExtRamp first removes the start and stop codons. Then the algorithm walks over each gene, codon by codon, and matches the associated tAI to each codon, creating an ordered list of codon efficiencies within that gene.

**Figure 2.**
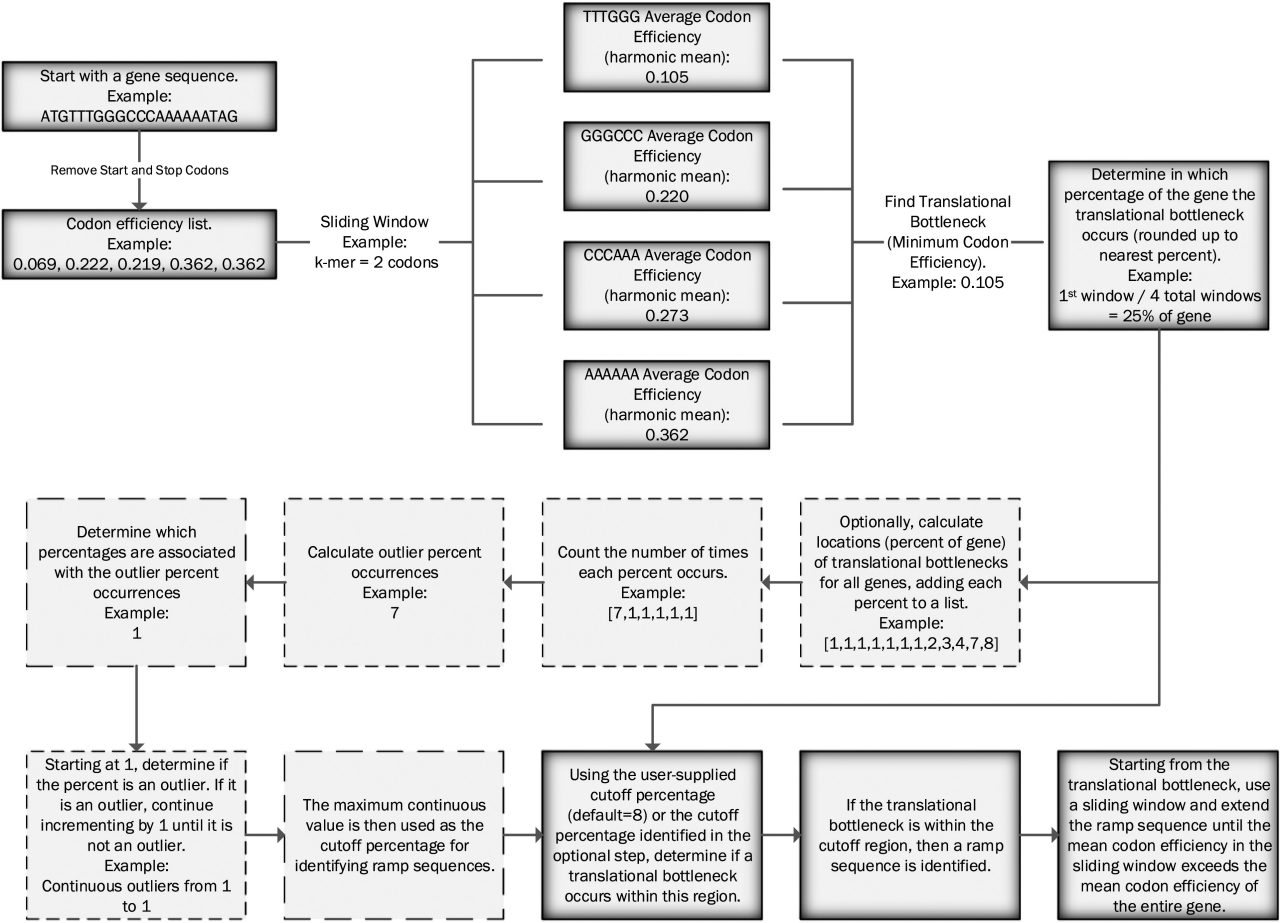
Translational bottleneck calculation and usage. A detailed example of how translational bottleneck outlier regions are calculated and used to identify ramp sequences.

Local codon efficiency bottlenecks are calculated by taking a sliding window the size of a ribosomal footprint (default nine codons (22)) of codon efficiencies, finding the middle (default harmonic mean) of each sliding window, and then determining where in the gene these bottlenecks occur, similar to the methods in Navon and Pilpel (23) (see Figure 2 for a detailed explanation with an example). Next, ExtRamp optionally determines regions across all genes in the input FASTA file where more local bottlenecks occur than expected by random chance (default is true outliers). Using this method, we take the most conservative approach to determine in which percentage of the gene the local minimum must occur for a ramp sequence to be identified by ensuring that all percentages (from 1 to n) are outliers (e.g. if 1, 2, 3, 5, 7 are outlier regions, then the local bottleneck must occur in the first 3% of the gene because 4 was not an outlier region). The user can specify outlier regions as well, in which case the bottleneck must occur within the user-defined outlier region. If a bottleneck occurs within this region, then the mean codon efficiency of the entire sequence is calculated. The ramp is extended beyond the bottleneck until the sliding window codon efficiency exceeds the mean codon efficiency of the whole sequence.

If the user specifies the arithmetic mean, geometric mean or median, the local translational bottleneck method outlined above is used as default. However, standard deviations can be used instead of local bottlenecks. If standard deviations are used, then the mean and standard deviation of all codon efficiencies within the sequence are calculated. Using a sliding window starting from the beginning of the gene, the ramp sequence extends until the mean codon efficiency within the sliding window exceeds the mean codon efficiency of the entire gene sequence minus the standard deviations specified by the user. However, since codon efficiencies have a large degree of variance and the sequences are relatively small, typically standard deviations must be smaller than 1.0 in order to identify ramp sequences.

An optional quality control step ensures that reported ramp sequences have similar lengths by calculating the average ramp length across all identified ramp sequences and removing ramp sequences that are in the tailing regions outside of a user-defined number (recommended two) of standard deviations above or below the mean length. Each step is multithreaded and by default uses all available processing cores, although any number of processing cores can be specified by the user.

The second option is used when the user does not supply the codon efficiency values for the species (i.e. the tAI values are not available for the species). This option uses either the input FASTA file or a user-supplied input FASTA file (typically containing highly expressed genes) to calculate the RSCU for each codon using the following formula, where *x*_*ij*_ is the occurrences of the *j*th codon in the *i*th amino acid, and *n*_*i*_ is the number of alternative codons for the *i*th amino acid (14):

(1) \begin{equation*}{{\rm RSCU}_{ij}} = \frac{{{x_{ij}}}}{{\frac{1}{n}\mathop \sum \nolimits_{j{\rm{\ }} = {\rm{\ }}1}^{{n_i}} {x_{ij}}}}\end{equation*}

Next, *w*_*ij*_ is calculated using the following formula:

(2) \begin{equation*}{w_{ij}} = \frac{{{{\rm RSCU}_{ij}}}}{{{{\rm RSCU}_{{\rm imax}}}}}\end{equation*}

These ratios estimate codon adaptiveness for a species, with smaller ratios associated with less adaptive (efficient) codons. Once these efficiencies are calculated, the analysis is the same as the tAI method, with w_ij_ substituting the tAI values. This second method extends the utility of ExtRamp to non-model organisms that are not yet included in the tAI library.

### Program options

ExtRamp is written in Python 3.5 and requires a few standard libraries which can easily be installed using pip3 (process outlined in the GitHub README). To increase the versatility of ExtRamp, we include several options that can be split into two categories: controlling input and output files, and specifying variables used in the algorithm. To see real-time progress of the algorithm at runtime, the -v (verbose) option can be used.

An input FASTA file with CDS sequences is required using the -i option. By default, DNA sequences are expected, although RNA can be provided using the -r flag. An optional input file containing tAI values (or other codon efficiency values) for each codon can also be provided using the -a option. If tAI values are not provided, ExtRamp will calculate ramp sequences based on *w_ij_* for the input FASTA file. However, *w_ij_* can be calculated on a different FASTA file using the -u option. By default, ramp sequences are printed to standard out (terminal) in FASTA format to facilitate piping the results into additional analysis tools. To print the ramp sequences to a file, the -o option can be provided. The list of local translation efficiencies for each sequence can be printed to a CSV file using the -l option. Each of the efficiency sequences are smoothed using the ribosomal window length (discussed below) and the data are printed in ‘tidy’ format (24) for easy graphing using R. An unsmoothed list of all codon efficiency speeds for each codon can also be written to a file using the -p option. A list of the gene names that did not contain any calculable ramp sequence can be written to a text file using the -n option and sequences that are removed because they are not divisible by three or do not exceed the minimum sequence length can be written to another file using the -z option.

There are nine options that control variables used in the analysis performed in ExtRamp. The -t option controls the number of threads used, with the default being all available processing cores. The -q option sets the minimum length of a sequence to be analyzed. Similar to the methods used by Navon and Pilpel (23), the default is 300 nucleotides (100 codons). Since there are several methods to determine the middle of a dataset, we provide the -m option with inputs of mean (arithmetic mean), median, gmean (geometric mean), and the default of hmean (harmonic mean). The -s option controls the number of standard deviations below the average of the consensus codon efficiency list for the maximum codon efficiency within a ramp sequence (typically less than one because codon efficiencies have large variances). The -d option controls the number of standard deviations above or below the mean ramp sequence length for all reported ramp sequences (if used, we recommend two standard deviations). The -w option controls the ribosomal window length that is used to smooth the proposed ramp sequences to minimize excess noise from spikes and dips in individual codon efficiencies. The default ribosomal window length is nine codons. The -f flag determines the outlier bottleneck regions based on sequences included in the input FASTA file. By default, the -f flag finds true outliers in the dataset. However, this can be modified using the -e option to find regions above a percentile (e.g. 75 would find places in a gene that have bottlenecks in the 75th percentile or above). The -c option sets the outlier region percentage (e.g. 10 would mean that the bottleneck must occur in the first 10% of the gene sequence). The default outlier region is in the first eight percent of the gene sequence.

### Algorithm validation

To validate our approach, we compared the consensus efficiencies calculated by ExtRamp for *S. cerevisiae, D. melanogaster*, and *C. elegans* to results by Tuller *et al.* (9). We used the tAI values published in that study rather than updated values to enable accurate comparisons. We found the consensus efficiency for each species using the ExtRamp algorithm and graphed the results. We ran the algorithm using the -m mean option to match the method used by Tuller *et al.* (9). The local efficiency values were also smoothed with a window size of four for consistency with their methods.

### FlyBase comparison

**Figure 3.**
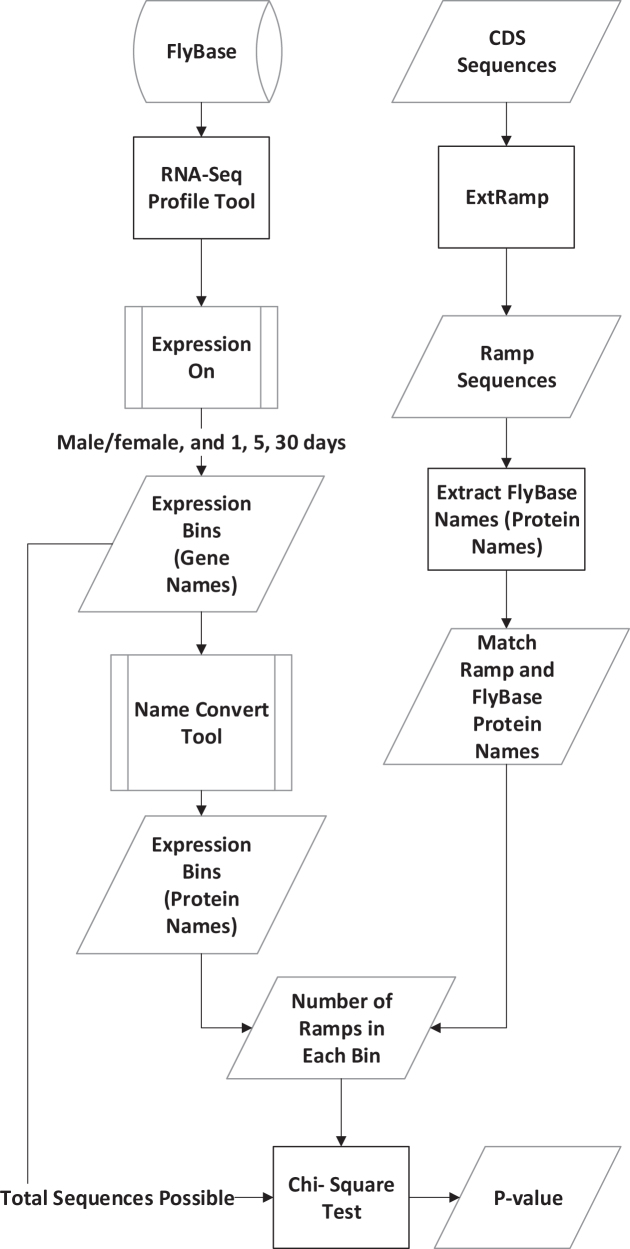
FlyBase analysis flowchart. Data were collected from both the FlyBase database and by running ExtRamp on *Drosophila melanogaster* coding sequences. The number of ramps that fell into each expression level bin was tested with a Chi-squared test to determine if the distribution was random.

We used RNA-Seq gene expression values reported in FlyBase (http://flybase.org/rnaseq/profile_search) (25) to determine if reported ramp sequences were associated with gene expression values (see Figure 3). We combined all expression data from both males and females at 1, 5 and 30 days old. Using the ‘Expression On’ utility, we pulled the FlyBase gene names for each of the eight expression level bins: ‘No/Extremely low’, ‘Very low’, ‘Low’, ‘Moderate’, ‘Moderately high’, ‘High’, ‘Very high’ and ‘Extremely high’. These gene names were converted to protein names using the provided FlyBase ‘convert’ tool to facilitate comparisons with our dataset. The RNA-Seq Profile tool uses a ‘not less than’ approach, so by default the ‘No/Extremely low’ bin contains all the genes that are identified by the higher expression bins as well. We ensured that each bin contained only genes with a certain expression level by removing all genes reported in bins with higher expressions.

We ran ExtRamp on the *D. melanogaster* CDS regions using the default options with tAI values. We then counted the number of ramp sequences for each expression level. Converting from gene names to protein names amplifies the number of sequences because there are multiple isoforms for each gene. Since we used the longest isoform of each gene, we used the number of gene names for the total number of sequences possible, instead of the number of protein names. Using a Chi-squared test, we checked if the number of hits for each expression level significantly differed from random.

### *w_ij_* versus tAI option comparison

To determine if running ExtRamp with and without tAI values produces similar results, we ran ExtRamp with and without tAI values on five species: *Acidilobus saccharovorans, Arabidopsis thaliana, C. elegans, D. melanogaster* and *S. cerevisiae*. We calculated the number of shared ramp sequences between the two techniques. We then used phyper, a mark and recapture statistical test built into R, to determine if the number of common elements was statistically significant. The following options were used: *p* = number of common sequences, *m* = number of tAI extracted sequences, *n* = total number of CDS tested – *m, k* = number of extracted sequences using w_ij_, and *lower.tail* = FALSE.

### Comparison across all domains of life

To further validate our approach, we used ExtRamp to extract ramp sequences from 229 742 339 gene sequences found in 23 428 species. We used the *w_ij_* method instead of tAI values for this analysis because tAI values are not available for most species. After extracting the ramp sequence from each gene, we determined the length of each ramp. For each species partition, we plot the percentage of genes with a ramp sequence and the length of the identified ramp sequences.

## RESULTS

**Figure 4.**
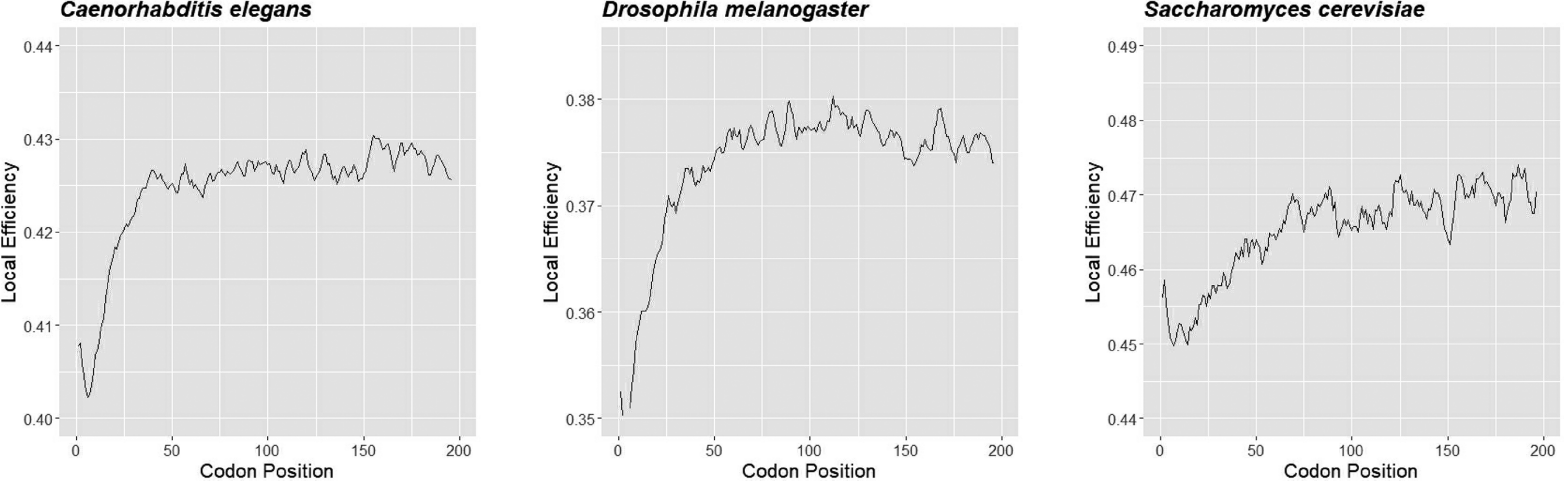
Consensus tAI Efficiencies: The averaged local tAI values across all CDS regions mapped to the codon position for *Caenorhabditis elegans, Drosophila melanogaster*, and *Saccharomyces cerevisiae*. The local efficiency values were smoothed with a window size of four. These graphs are identical to charts reported in Tuller *et al.* (9).

We first tested the accuracy of our algorithm by replicating the consensus translation efficiency of species reported in *Tuller et al.* Using parameters specified in their manuscript, ExtRamp reports identical codon efficiencies at each position (Figure 4).

**Figure 5.**
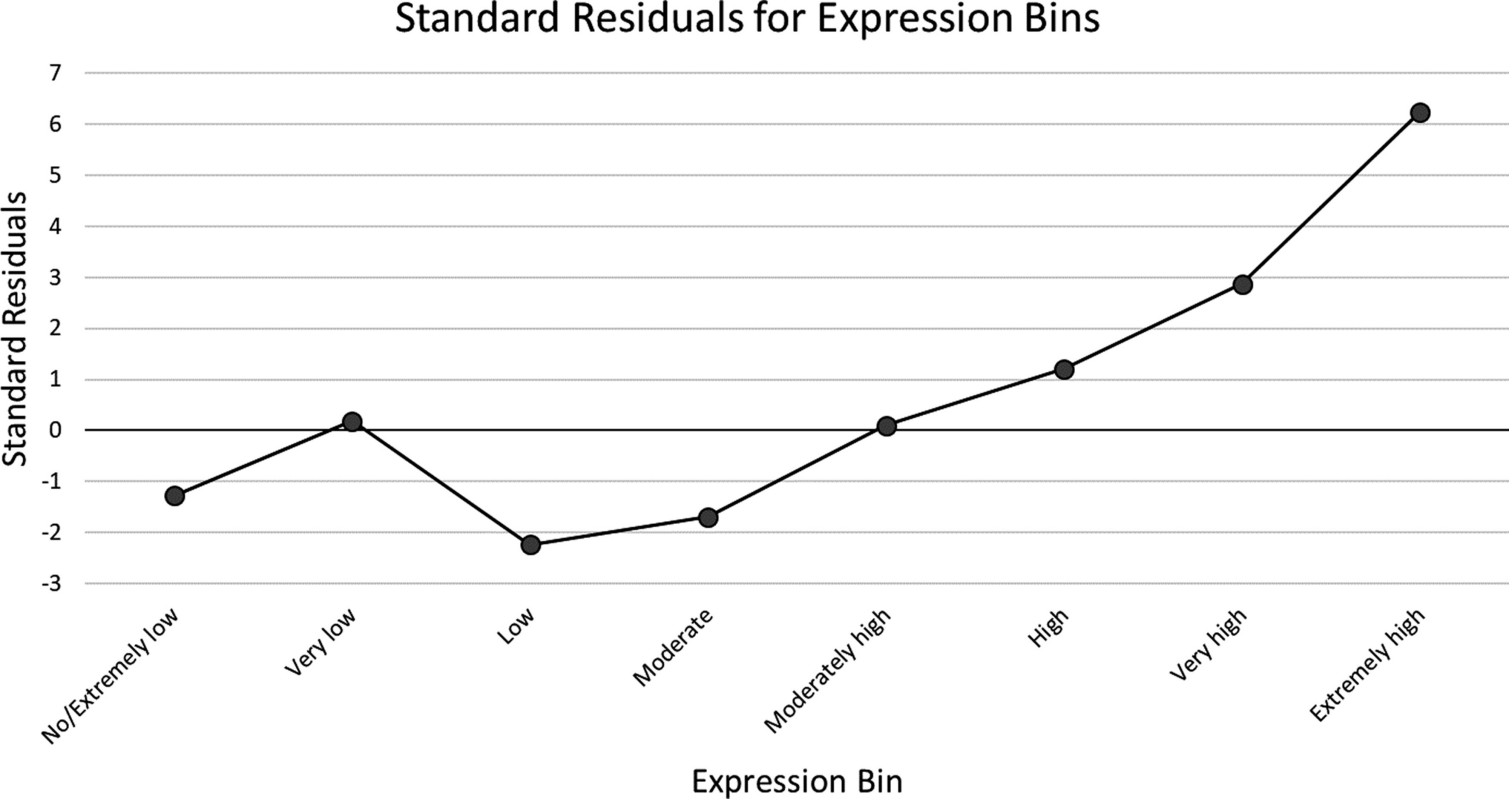
Standard residuals of expression bins. Using a Chi-squared test, we calculated the standard residuals for each expression bin and plotted these values, ordered from the bin with the lowest expression to the bin with the highest expression.

We then determined if ramp sequences were associated with gene expression values using the tAI method (Table 1). Using the detailed gene expression data available for *D. melanogaster*, we compared the isolated ramp sequences to their respective expression level bin. Using a Chi-squared test, we compared the number of genes found in each bin to the expected number if the ramp sequences were proportionally distributed between the expression bins. The reported Chi-squared value was 58.2 with seven degrees of freedom and a p-value of 3.45 × 10^−10^. A clear progression of increasing standard residuals is seen from genes with low expression to extremely high expression. Very low and extremely low expression genes have slightly higher standard residuals than low expression genes (0.19 and −1.28, respectively). However, the residuals are much lower than very high and extremely high expression genes (2.87 and 6.24, respectively). We plot the standard residuals in Figure 5 to show the trend toward more ramp sequences in more highly expressed genes in *Drosophila*.

**Table 1.** tAI Ramp sequences for FlyBase expression bins

Expression level	Observed ramps	Total sequences	Expected ramps	Standard residuals
**No/Extremely low**	73	726	84.82014734	−1.28
**Very low**	182	1536	179.454196	0.19
**Low**	181	1830	213.8028507	−2.24
**Moderate**	337	3162	369.4232864	−1.69
**Moderately high**	312	2655	310.1893818	0.1
**High**	177	1383	161.5788757	1.21
**Very high**	155	1054	123.1410955	2.87
**Extremely high**	42	142	16.59016656	6.24
**Total**	1459	12488	1459	

Ramp sequences were extracted from all genes reported in FlyBase using ExtRamp and tAI values. For each expression level (no/extremely low to extremely high), the number of observed ramp sequences was compared to the expected number of ramp sequences if ramp sequences were not associated with expression (i.e. the total proportion of ramp sequences multiplied by the total number of sequences in a bin reported by FlyBase).

We compared the *w_ij_* and the tAI approaches to identify ramp sequences. The number of ramp sequences extracted from each species varied between these approaches, so we calculated if the number of common sequences between the approaches was random or if both options were targeting the same sequences. Using a Mark and Recapture statistical approach on five species, four of the five species had very significant p-values (<1 × 10^−6^), indicating that the two approaches typically identified ramp sequences for the same genes (Table 2).

**Table 2.** Mark and recapture analysis: the number of ramps extracted using the tAI and *w_ij_* options was determined from the total number of gene sequences

Species	Number of ramps identified tAI method	Number of ramps identified *w_ij_* method	Number of total sequences	Number of identical ramps captured	Mark and recapture *P*-value
***Arabidopsis thaliana***	1974	3672	25101	239	0.999
***Acidilobus saccharovorans***	191	261	1354	63	2.95 × 10^−7^
***Caenorhabditis elegans***	3294	2897	18901	640	9.78 × 10^−13^
***Drosophila melanogaster***	1848	2302	12920	850	2.21 × 10^−210^
***Saccharomyces cerevisiae***	823	767	5649	256	1.66 × 10^−47^

The ‘number of identical ramps captured’ indicates the number of sequences that contained ramps using both tAI and wij methods of ramp extraction. The p-value indicates the probability that the amount of overlap (‘number of identical ramps captured’) could occur randomly. The phyper function in R was used for these calculations.

**Figure 6.**
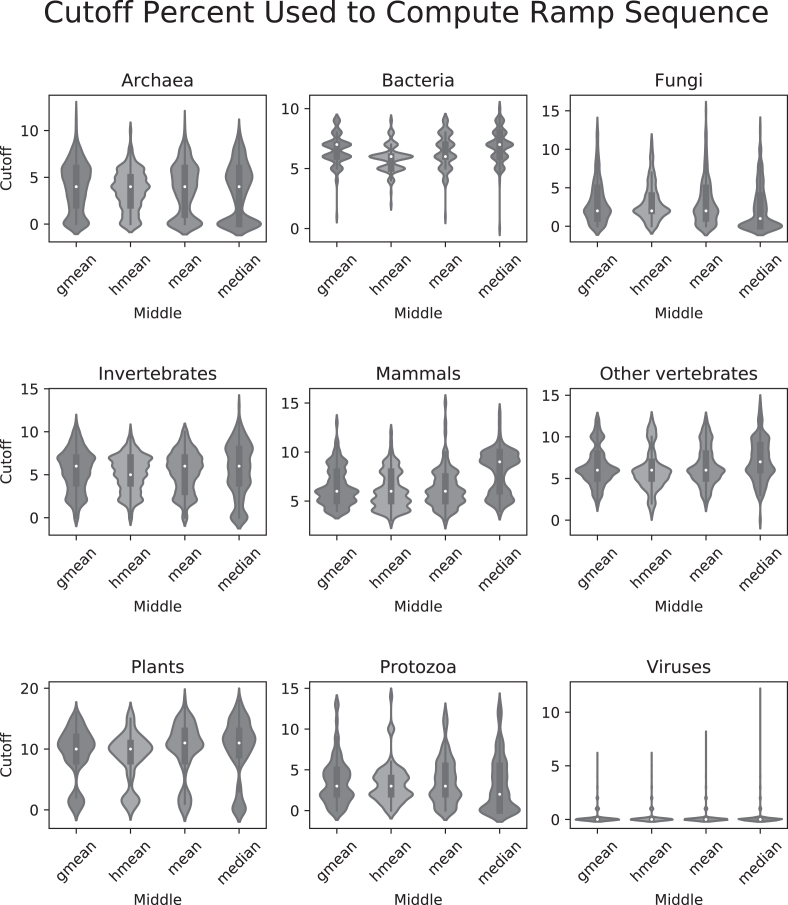
Cutoff percentage used to compute ramp sequence. For each taxonomic group, violin plots for each of the four middle values show the cutoff percentages used to compute the ramp sequences. Cutoff percentages are defined as the last consecutive gene region before the number of translational bottlenecks is no longer an outlier, starting from the first percentile (i.e. if the cutoff is 5, then 1–5 are all outlier regions). Each of the nine subplots show means in the following order: geometric mean, harmonic mean, arithmetic mean and median.

**Figure 7.**
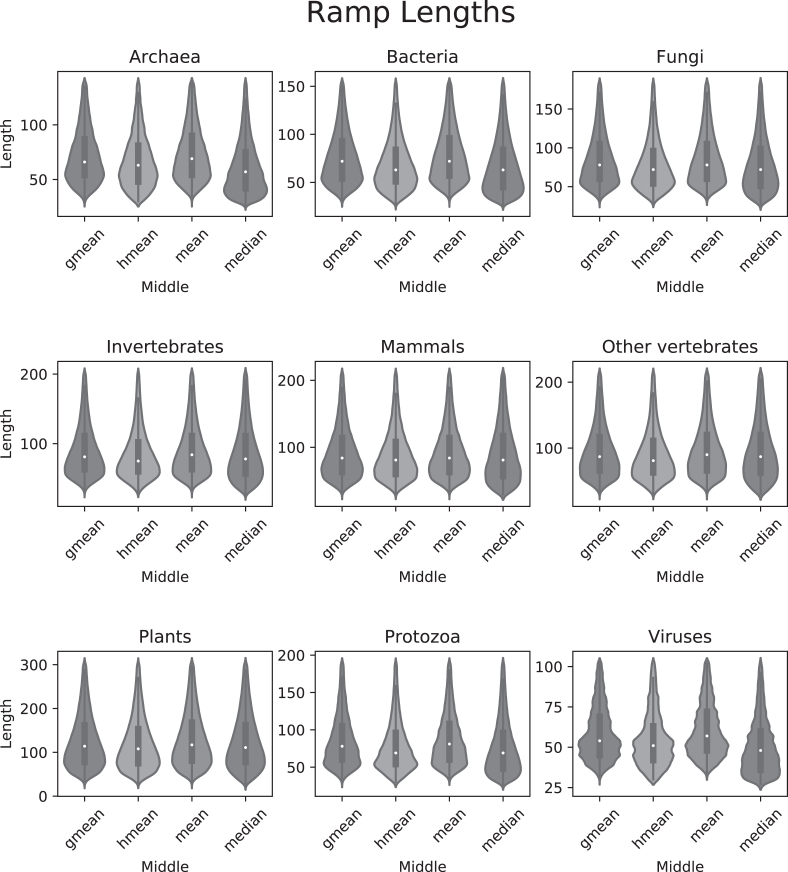
Ramp lengths in nucleotides. After removing outliers, we plot the ramp lengths for all ramp sequences in each taxonomic group. Each of the nine subplots show means in the following order: geometric mean, harmonic mean, arithmetic mean and median.

**Figure 8.**
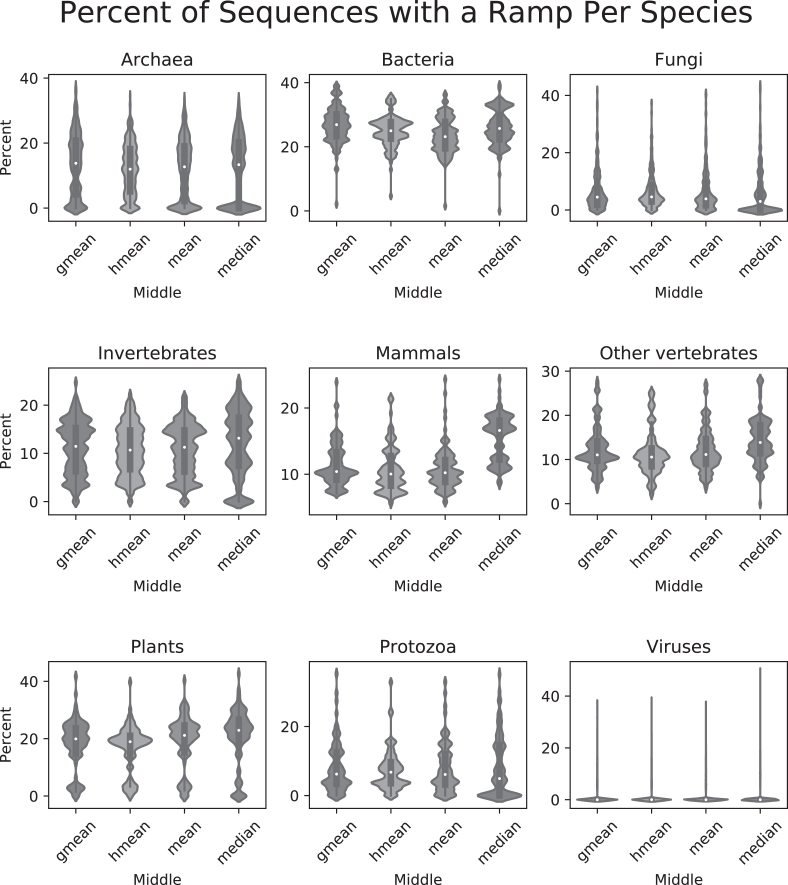
Percentage of sequences with a ramp per species. For each taxonomic group, violin plots for each of the four middle values show the percent of sequences in each species that contained a ramp. Each of the nine subplots show means in the following order: geometric mean, harmonic mean, arithmetic mean and median.

Finally, we identified ramp sequences in all genes from 23 428 different species across all domains of life using the *w_ij_* method. In all instances, similar ramp sequences were reported using any of the four middle values: geometric mean, harmonic mean, arithmetic mean, and median. The first 5–10% of the gene was typically considered an outlier region, with protozoa having a slightly lower average (2–5%) and viruses reported almost no outlier regions (see Figure 6). Reported ramp lengths in sequences with a ramp typically ranged from about 60 to 120 nucleotides (20–40 codons), with plants having a slightly higher average length (about 25–55 codons) and viruses having a slightly lower average length (about 10–20 codons) (see Figure 7). Bacteria and plants reported the highest percentage of genes with ramp sequences (15–30%), while viruses reported almost no genes with ramp sequences (see Figure 8). Using the translational bottleneck technique with the strictest filter for outliers, most taxonomic groups report ramp sequences for about 10% of all species genomes (Figure 8).

**Figure 9.**
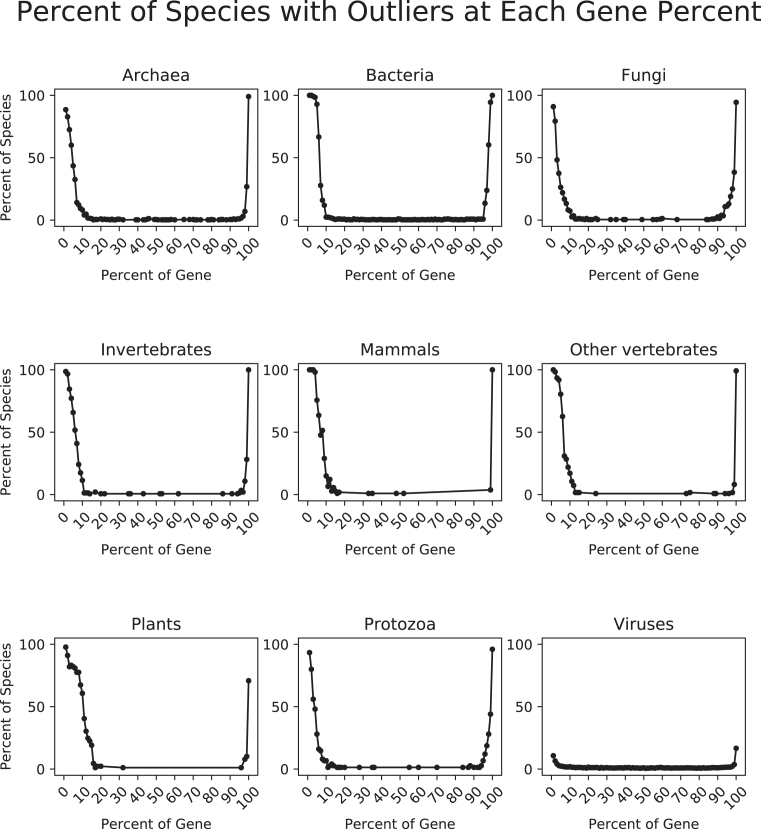
Percent of species with outliers at each gene percent. After dividing each gene into 100 equal parts, we determined where translational bottlenecks occur in the gene. We then identified all outlier regions using the harmonic mean. For each taxonomic group, we counted the number of species with an outlier region at each of the 100 percentiles, and we divided that number by the total number of species in the taxonomic group. We plot these percentiles. When no species had an outlier region, points are not plotted.

We also analyzed the outlier regions that were identified by ExtRamp. Since all middle values report similar ramp sequences, we chose the default harmonic mean to analyze the outlier percentiles. We first removed the start and stop codon from each gene in the genome. Then we divided each gene into 100 equal parts and determined in which part the translational bottleneck occurred. Where multiple equal bottlenecks were identified, all bottlenecks were included in the analysis. We show that in bacteria, invertebrates, mammals, other vertebrates, and plants, 100% of the species had an outlier region in the first percentile of their genes, and the outlier region extends to the tenth percentile in most taxonomic groups (see Figure 9). We also found that all taxonomic groups have an outlier region in the last percentile (99th percentile) of the gene. Each taxonomic group except viruses clearly shows an outlier region at the beginning of the gene sequence with very few outlier regions between the first 10% of the gene and the end of the gene.

## DISCUSSION

Using the strictest settings on ExtRamp, most taxonomic groups had similar percentages of ramp sequences (∼10% of genes) and ramp sequence lengths (20–40 codons). However, bacteria and plants reported significantly more ramp sequences (∼25% of genes), while viruses reported almost no genes with a ramp sequence. In plants, the reported cutoff value was higher than the other taxonomic groups (Figure 6), indicating the outlier regions extended farther into the gene sequence. More outlier regions could indicate that in plants, translational bottlenecks occur in a wider region at the beginning of gene sequences than in other taxonomic groups. A higher reported cutoff percentage would increase the number of ramp sequences identified and could account for the increased number of ramp sequences shown in Figure 8. In bacteria, the mean cutoff percentage is slightly higher than in other taxonomic groups. However, the density distribution is much more tightly concentrated around the mean (see Figure 6). A tighter density distribution indicates that bacteria report more similar cutoff percentages between species than inter-species comparisons in other taxonomic groups. This tighter density distribution could also indicate that more selective pressure exists in bacteria to maintain a ramp sequence than in other taxonomic groups. Unsurprisingly, viruses reported almost no ramp sequences. Viral genes are populated with regulatory sequences at the beginning of the coding region and host-repeating substrings throughout the genetic code (26), which potentially limits the applicability of selection for ramp sequences in viruses.

We also present evidence that a clear progression of increasing proportions of ramp sequences are identified from low expressed genes to extremely high expressed genes in *D. melanogaster* (Table 1). We plot the standard residuals for each expression bin (Figure 5) and show that the highest standard residual (6.24) is found in the ‘Extremely high’ expression bin, while all expression bins less than or equal to ‘Moderately high’ expression have standard residuals at or below zero. This analysis complements previous studies indicating that ramp sequences are more prevalent in highly expressed genes (9,27).

Although the *w_ij_* and tAI methods detect different numbers of sequences as containing ramps, they largely target the same sequences, with four of the five species analyzed having *p*-values <1 × 10^−6^ (Table 2). Since tAI values are not available for most species, further evaluation with a more robust tAI library might indicate systematic biases of tAI, whether from a phylogenetic or algorithmic standpoint. It is probable that tAI is more accurate in some species, or the correlation between tAI and *w_ij_* is not universal. However, through our analysis, we show that although *w_ij_* and tAI recover different numbers of ramp sequences, both methods typically target the same sequences.

ExtRamp can also aid in the analysis of a gene as a whole. Because the ramp sequences behave differently than the rest of the gene, it can skew the results of certain analyses. Some studies have avoided the problem by removing the first 50 codons of all the sequences before performing their analyses (28). However, this practice removes potentially valuable data and is not universally accurate for all sequences or species. At least two solutions to this predicament are as follows: (i) Determine the exact ramp sequence for each gene (possibly none) and remove only those portions, thereby keeping more of the sequence data for downstream analysis. (ii) Incorporate the annotated ramp sequences in the downstream analysis tools.

We also provide the option to view the local translation efficiencies for each sequence that can easily be plotted using R. With these data, analyses can extend beyond the ramp sequence into the body of the gene. Furthermore, the option to view codon efficiency at each position allows for more extensive analyses involving local translational bottlenecks and codon usages. Future analyses could evaluate if there are correlations between physical characteristics such as functional domains of the gene and the translational efficiency of that section of the gene.

Very few studies have been performed on ramp sequences because software for extracting individual ramp sequences does not exist. We developed this algorithm to fill this need and improve the study of ramp sequences. Many studies look at ramp sequences on a high level, either evaluating the average length of the sequences in a species or determining the codon usage bias that influences the ramp. ExtRamp is the first algorithm to isolate the ramp sequence from individual genes, and it is the first attempt to analyze ramp sequences in non-model organisms. Future research can determine which codons, specifically, are targeted in the ramp sequence, if ramps have a different mutation rate than the rest of the gene, if ramp sequences are associated with DNA structure, and if the length of the ramps can be used as a predictor for expression levels. We anticipate that ExtRamp will make ramp sequence research more accessible and assist in uncovering more biologically meaningful interpretations of the ramp sequence.

## DATA AVAILABILITY

ExtRamp is an open source collaborative project available in the GitHub repository (https://github.com/ridgelab/ExtRamp).
